# Evaluation of the efficacy of modal analysis in predicting the pullout strength of fixation bone screws

**DOI:** 10.1002/jsp2.1220

**Published:** 2022-10-19

**Authors:** Mohammadjavad Einafshar, Ata Hashemi, Ali Kiapour

**Affiliations:** ^1^ Biomechanical Engineering Group, Department of Biomedical Engineering Amirkabir University of Technology Tehran Iran; ^2^ Department of Material and Production Aalborg University Aalborg Denamrk; ^3^ Departments of Neurosurgery Massachusetts General Hospital, Harvard Medical School Boston Massachusetts USA

**Keywords:** bone‐screw interface, insertion torque, modal analysis, natural frequency, pilot hole diameter, primary stability, pullout strength

## Abstract

**Background:**

Pilot hole preparation has been shown to have an impact on the short and long‐term stability of the screw fixation constructs.

**Purpose:**

Investigation and comparison of two nondestructive modal analysis methods with conventional insertion torque (IT) and pullout tests in optimum pilot hole diameter detection.

**Methods:**

Twenty conical core titanium screws were embedded in high‐density polyethylene blocks with different pilot hole diameters. The maximum IT was recorded for each screw during implantation. Then, two modal analysis methods including accelerometer (classical modal analysis [CMA]) and acoustic modal analysis (AMA) were carried out to measure the natural frequency (NF) of the bone‐screw structure. Finally, stiffness (S), pullout force (F_ult_), displacement at F_ult_ (d_ult_) and energy dissipation (ED) were obtained from the destructive pullout test.

**Results:**

The IT increased, as the pilot hole diameter decreased. The maximum value of IT was observed in the smallest pilot hole diameter. The same trend was found for the F_ult_ and the first NF derived from both modal methods except for 5.5 mm pilot hole diameter. The natural NFs derived from CMA and AMA showed high correlations in different groups (R^2^ = 0.94) and did not deviate from y = x hypothesis in linear regression analysis. The F_ult_, d_ult_, and ED were measured 4800 ± 172 N, 3.10 ± 0.08 mm and 14.23 ± 1.10 N.mm, respectively.

**Discussion:**

No significant change was observed in “S” between the groups. The highest F_ult_ and first NF were obtained for the 5.5 mm pilot hole diameter. Both CMA and AMA were found to be reliable methods and can promote the undesirable contradiction between F_ult_ and IT.

## Graphical abstract

We evaluated the efficacy of a novel method in predicting the pullout strength of bone screws. The analysis showed that the pullout strength is highly affected by the size of the pilot hole. The nondestructive method can be used to predict the pullout strength of the bone screws.

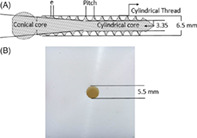

## 1 INTRODUCTION

Bone screws are widely used as an internal fixation component for various bone fractures,^1^ deformities,^2^ fixation,^3^and implant fragments.^4^ The success of internal fixation has been found to depend mainly on the implant primary stability that occurs immediately after the implant insertion.^5^

Insertion of screws into the bone often requires preparation of the anatomical structure of the intended bone through creation of the pilot hole. The pilot holes are often created using static or punch awls or drills.^6^ The diameter of the pilot hole should be in proportion to the diameter of the bone screw to ensure adequate mechanical stability of the screw in the bone.^7^

Researchers have reported that the holding power of the screw depends on the changes induced in bone by the insertion trauma as well as bone reaction around the implant. It is also known that the fixation of the screw in the bone depends on material characteristics of the surrounding bone,^8^ the implant design,^9^, ^10^, ^11^, ^12^ the pilot hole size and the preparation technique.^6^, ^13^, ^14^ The press fit between the screw and the bone is related to the pilot hole size, affecting the quality of the implant fixation.^15^ Most of the studies have used in vitro experimental tests to investigate the influence of the pilot hole size on insertion torque (IT) and pullout force of the synthetic bone,^6^, ^7^, ^16^, ^17^, ^18^ however, a few studies were performed on human bone.^17^, ^19^, ^20^, ^21^

The effects of multiple and repetitive reinsertions on pullout strength have been evaluated in several studies, however, the results were contradictory in some cases.^14^, ^22^ A decrease of 13%, 33%, and 33% in pullout force was measured when the 5.5 mm bone screws were inserted into polyurethane foam, polyethylene and bovine samples, respectively, in the study of Defino et al.^14^ Some studies investigated the pilot hole preparation by using cement augmentation and cannulated screws.^23^ Boyle et al. reported an increase in pullout strength and IT in higher differences between the outer diameter of screw threads and drill size.^24^ Heidemann et al. indicated that the pilot hole size could increase up to 85% of the screw outer diameter, without affecting the pullout force.^13^ Moreover, the difference between screw thread diameter and drill size was shown to affect the plastic deformation and maximum radial deformation of the pilot hole.^25^The prediction of pullout strength was found to be more realistic when the effects of plastic deformation were applied by Einafshar et al.^18^, ^26^ The above studies mainly rely on the use of IT and the pullout strength to evaluate the effects of the pilot hole size variations. Nevertheless, a weak correlation between the pullout strength and insertion torque values has been the focus of other studies.^27^, ^28^

New techniques such as modal analysis method have been recently introduced for the fixation strength assessment of dental implants,^29^, ^30^ hip prostheses^31^, ^32^and spinal pedicle screws.^33^, ^34^, ^35^, ^36^, ^37^, ^38^ This method has yielded a new approach to evaluate both primary and secondary stability of dental implants.^5^ The nondestructive modal analysis technique has been used to determine the initial stability of spinal pedicle screws by extracting the fundamental natural frequency (NF) from recorded acceleration‐time response.^38^, ^39^

To further investigate the applicability of the newly introduced modal analysis method, the present study was aimed to evaluate the effect of different pilot hole diameters on the primary stability of pedicle screws in the high‐density polyethylene (HDPE). The primary stability was measured with four different methods in order to verify the accuracy and reliability of the methods. The results of both modal analysis methods that is, classical modal analysis and acoustic one were compared with the conventional pullout strength and IT tests to investigate the optimum pilot hole diameter.

## 2 MATERIAL AND METHODS

### 2.1 Pedicle screw specification

**FIGURE 1:**
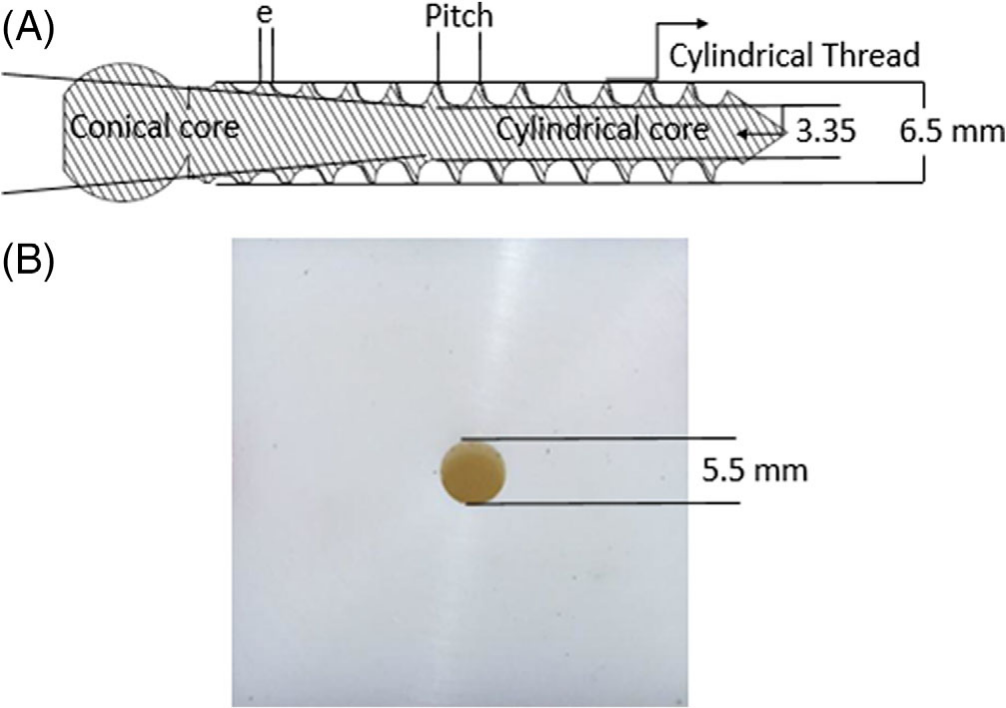
(A) Schematic of the pedicle screw and (B) high‐density polyethylene used in this study

Twenty titanium, self‐taped, cylindrical thread and conical core pedicle screws (Fortex™, XTANT Medical, MT, USA) used in our previous work, were utilized in this study.^18^ The thread pitch was constant throughout the screw and the crest thickness was reduced from the proximal to the distal portion, to improve bone integrity with both the cortical and cancellous regions.^8^ The thread shape changed gradually from triangular to squared type from distal to the proximal part of screw (Figure 1).The squared shape of the cylindrical thread and the conical core caused a better grip at the bone and screw interface in both the cortical and cancellous regions.^8^, ^40^ Accordingly, the core diameter increased from 3.35 to 5.35 mm throughout the screw. The length and thread diameter of the screw were 47 and 6.5 mm, respectively.

### 2.2 Block test

The HDPE (Direct Plastics Ltd., Sheffield, UK)block was cut into the samples of 40 × 40 × 60 mm^3^ (Figure 1). The Young's modulus and density of HDPE were 1000 MPa and 947 kg/m^3^, respectively. A total number of 20 HDPE blocks (*N* = 5/group) were used for screw insertion. The HDPE is uniform, nonporous, homogenous, bone‐like properties and machinable. All these together make HDPE providing creation of accurate pilot hole diameter that is, 0.25 mm accuracy between groups.

### 2.3 Insertion of screw

**FIGURE 2:**
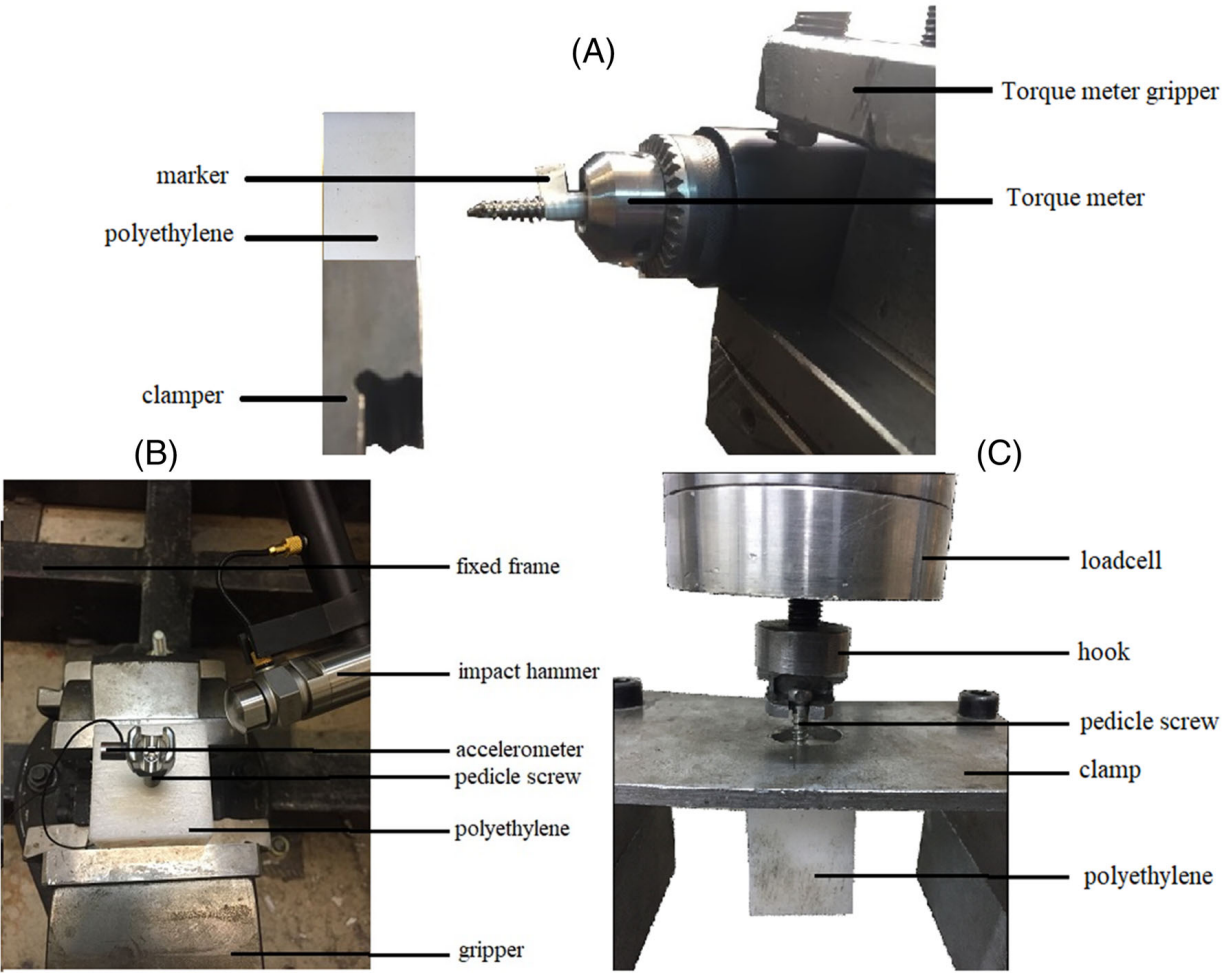
The setups for (A) screw insertion using a digital torque meter, (B) modal analysis, and (C) pullout test

The pilot hole diameter was adjusted to be slightly larger than the core diameter base on the manufacturer's instructions and under provision of ASTM F543.^41^ In the present study, four different pilot hole diameters of 5, 5.25, 5.5, and 5.75 mm (*N* = 5/group) were chosen to investigate the effect of pilot hole diameter. These dimensions are the final pilot hole diameters. The pilot hole was drilled into the depth of 20 mm, as suggested by ASTM F543.^41^ The pilot hole drilling parameters have remarkable effects on results^42^, ^43^ and all drilling parameters including feed rate and spindle speed kept constant during the tests. The pilot hole diameters of 5 and 5.25 were considered slightly smaller and diameters of 5.5 and 5.75 were considered a bit larger than the major core diameter of the screw. Bone screws were inserted into the HDPE samples using a torque meter (TQ‐8800, LT Lutron, Taiwan) to achieve desired insertion depth (Figure 2A). Furthermore, the peak IT (PIT) was recorded for each test.

### 2.4 Modal analysis

#### 2.4.1 Classical modal analysis

Classical modal analysis (CMA) was performed for each sample very similar to the previous studies.^38^, ^39^ A unidirectional accelerometer (code of 4374, Bruel & Kjaer Inc., Denmark) with 0.27 g mass was attached to the flattened head of the screw using a thin double‐sided tape (Figure 2B). The recording device was calibrated using multiple impacts to avoid overloads. A standard impact hammer (code of 8202, Bruel & Kjaer Inc., Denmark) was used to tap the head of the screw horizontally. The accelerometer time response was recorded and analyzed. Damped NF (ω_d_) was measured using the recorded acceleration‐time response curve. Decaying oscillation amplitude (δ) was then calculated using Equation 1 where amplitudes of two sequential periods in the acceleration‐time response curve are indicated as a (*t*) and a (*t* + *T*). Next, the system's damp ratio (ζ) and the NF (ω_n_) were determined from Equations 2 and 3, respectively.^38^, ^44^ This step of the test was repeated four times for each sample. A number of 20 modal tests were carried out for each pilot hole diameter; 80 modal tests in total for CMA.

(1) $$\delta =\ln\left(\frac{A\left(t\right)}{A\left(t+T\right)}\right)$$

(2) $$\zeta =\frac{\delta }{\sqrt{4\pi^{2}+\delta^{2}}}$$

(3) $$\omega_{n}=\frac{\omega_{d}}{\sqrt{1-\zeta^{2}}}$$

#### 2.4.2 Acoustic modal analysis

During the tests, the tapping sound was simultaneously recorded using a microphone(Audio–Control CM‐145, Apple Inc. USA) attached to the accelerometer. The recorded sound was transferred to MATLAB R2017 (Natick, Mathworks Inc. USA) software and data converted from time domain into the frequency domain using fast Fourier transform. The location of the peaks illustrated the natural frequencies at different modes.^38^, ^39^ The first peak in the frequency response is known as the first mode of vibration.^44^ In addition, by detaching the accelerometer, the acoustic modal analysis (AMA) was repeated for all samples four times (80 modal tests with an accelerometer attached and 80 without it) to determine the NF of the screw‐block setup, without the accelerometer and examine the effect of added mass. Furthermore, the results were compared with the pullout and IT tests.

### 2.5 Pullout test

The pullout test was performed (*N* = 5/pilot hole diameter) according to the ASTM‐F543 very similar to the previous study,^18^ using a uniaxial tensile testing machine (Zwick/Roell, DTM, Germany). After the placement of the pedicle screw within the HDPE block, the orientation of the pedicle screw and tensile hook adjusted to the coaxial orientation and then the load cell set to zero. The displacement rate was 5 mm/min, data collection continued at a rate of 25 Hz until complete pullout of the screw (Figure 2C). pullout characteristics that is, stiffness (S), peak pullout force (F_ult_), displacement at F_ult_ (d_ult_) and dissipated energy (DE) were also determined for each pullout test.

### 2.6 Statistical analysis

Linear regression analysis was used to assess the efficiency of the CMA and AMA methods in the prediction of the NF. Statistical differences between the resulting regression line and the slope intercept of the y = x line were also undertaken. One‐way ANOVA with a confidence level of 95% (*p* < 0.05) was used to analyze the differences between the groups in terms of the IT, pullout characteristics and NF using Microsoft Excel (STATA, StataCorp, College Station, TX, USA). Furthermore, a Tukey–Kramer honesty significant difference (HSD) post hoc test was used to determine significant differences among the results in each test group.

## 3 RESULTS

**FIGURE 3:**
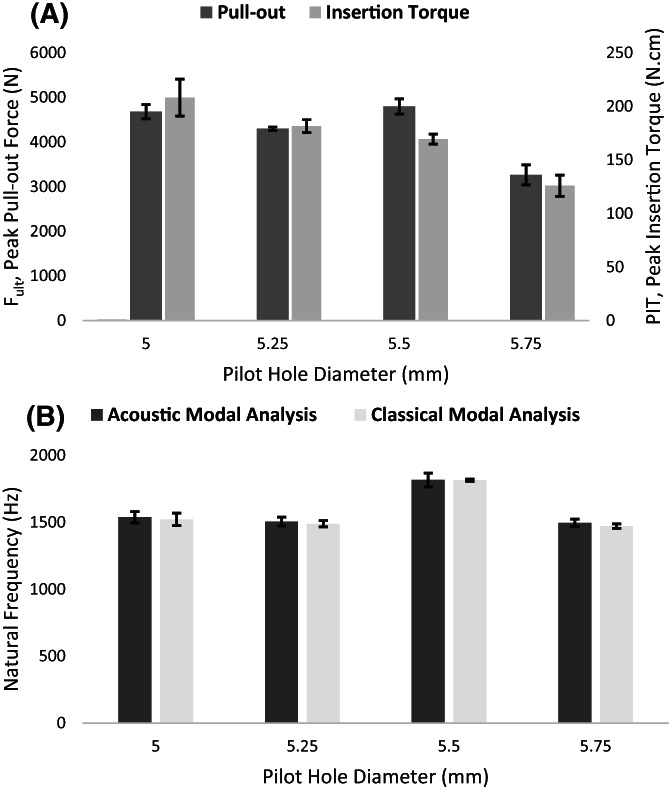
(A) peak pullout force (F_ult_) and peak insertion torque (PIT). (B) acoustic natural frequency and accelerometer natural frequency versus four different pilot hole diameters

**FIGURE 4:**
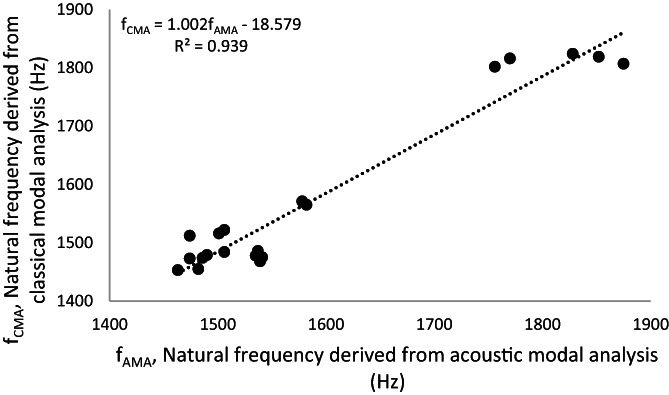
Linear regression analysis for both CMA and SMA methods that is, accelerometer natural frequency (f_CMA_) versus acoustic natural frequency (f_AMA_). Linear regression line passes through y = x line and did not deviate from zero intersect (*p* value >0.05)

The PIT decreased 65% by increasing of pilot hole up to 15%. The maximum and minimum PIT were 208 ± 16 and 126 ± 12 N.cm for 5 mm and 5.75 mm pilot hole diameters, respectively (Figure 3A). The maximum (1816 ± 52 Hz) and minimum (1495 ± 27 Hz) acoustic NF (f_AMA_)were obtained in 5.5 and 5.75 mm pilot hole diameters, respectively (Figure 3B). Similarly, the maximum and minimum accelerometer NF (f_CMA_) placed in 5.5 and 5.75 mm pilot hole diameters and the values for f_CMA_ were quantified 1813 ± 8 and 1471 ± 15 Hz, respectively (Figure 3B). A very high correlation between f_CMA_ and f_AMA_ was calculated (R^2^ = 0.94, *p* < 0.001) and the slope of the regression curve did not deviate from the y = x line (f_CMA_ = 1.002, f_AMA_−18.6) (*p* = 0.83) (Figure 4).

The F_ult_ decreased by 43% by increasing pilot hole diameter from 5 to 5.75 mm (Figure 3A). The maxima (4800 ± 172) and minima (3266 ± 225 N) F_ult_ were calculated for 5.5 and 5.75 mm pilot hole diameters, respectively (Figure 3A). Likewise, the maximum and minimum stiffness (S) were observed in 5 and 5.75 mm pilot hole diameter cases, respectively, where the gap between screw core and hole diameter was the smallest and largest among the groups, respectively. On the other hand, the maximum and minimum of DE were observed in 5.5 and 5.75 mm pilot hole diameters, respectively. Also, a decrease of 15% in hole diameter resulted in an increase in S and DE up to 17% and 72%, respectively (Table 2).

**FIGURE 5:**
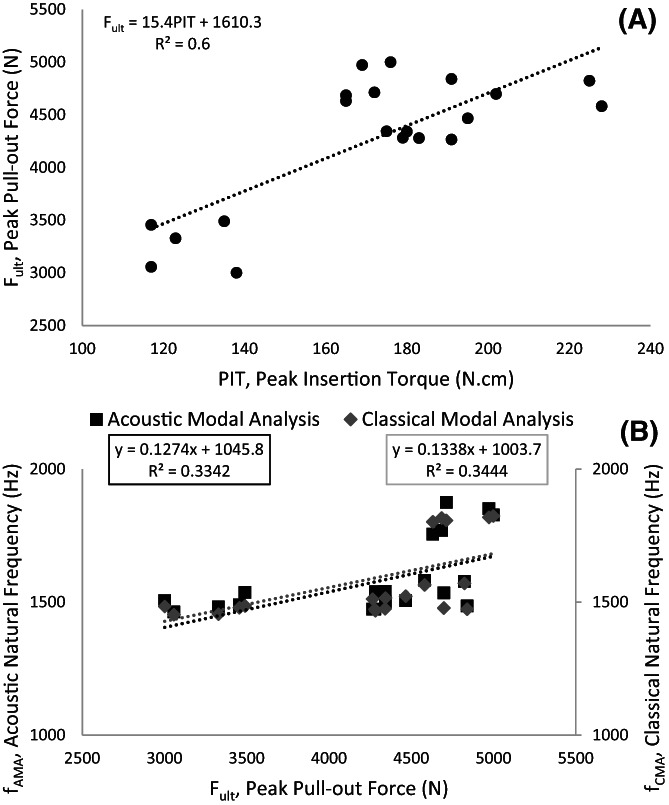
Linear regression analysis for (A) peak insertion torque (PIT) and peak pull‐out force (F_ult_). (B) acoustic natural frequency and classical natural frequency versus peak pullout force

The decreasing trend of PIT and F_ult_ were not the same (Figure 3A). The linear R‐square value of PIT and F_ult_ was calculated 0.61 (Figure 5A), while the R‐square value for linear trend in case of removing the 5.5 mm group was 0.66. The decreasing pattern was observed in dependent variables that is, the F_ult_ (Figure 3A), f_AMA_ (Figure 3B) and f_CMA_ (Figure 3B), except for the 5.5 mm pilot hole group, while the decreasing pattern was observed in PIT without any exception (Figure 3A).The one‐way ANOVA test results indicated that all groups within every dependent variable that is, the PIT, F_ult_, S, DE, f_AMA_, and f_CMA_ were significantly different (*p* < 0.001). The significant subgroups were examined by the HSD test (Table 1).

**TABLE 1:** Tukey–Kramer post hoc test for all subgroups

Groups	5	5.25	5.5	5.75
5	‐	PIT−f_ult_	PIT−f_SMA_−f_CMA_	PIT−f_ult_−f_SMA_−f_CMA_
5.25	PIT−f_ult_	‐	f_ult_−f_SMA_−f_CMA_	PIT−f_ult_
5.5	PIT−f_SMA_−f_CMA_	f_ult_−f_SMA_−f_CMA_	‐	PIT−f_ult_−f_SMA_−f_CMA_
5.75	PIT−f_ult_−f_SMA_−f_CMA_	PIT−f_ult_	PIT−f_ult_−f_SMA_−f_CMA_	‐

*Note*: PIT, f_ult_, f_SMA_, and f_CMA_ stand for, peak insertion torque, peak pullout force, sound, and classical natural frequencies, respectively.

Different characteristics of the pullout test that is, the S, F_ult_, d_ult_, and ED for 5 and 5.5 mm pilot hole diameters demonstrated similar patterns (Table 2) and all were significantly different in each group based on the ANOVA test (*p* < 0.001). According to the Tukey–Kramer test, the F_ult_, d_ult_, and ED were significantly different between all the subgroups, except between 5 and 5.5 mm pilot hole diameter, while “S” showed a different pattern and was significantly different between the three first pilot holes and 5.75 mm pilot hole diameter (Table 2).

**TABLE 2:** Peak pullout force (F_ult_), displacement at F_ult_(d_ult_), stiffness (S), and dissipated energy (DE), which were extracted form pullout test the two last rows showed one‐way ANOVA test overall results and Tukey–Kramer HSD test among the pairs, respectively

Group	Pilot hole diameter (mm)	S (N/mm)	F_ult_ (N)	d_ult_ (mm)	DE (N.m)
A	5.00	1910 ± 20	4682 ± 160	2.90 ± 0.11	14.21 ± 0.70
B	5.25	1863 ± 22	4302 ± 37	2.63 ± 0.08	11.52 ± 0.16
C	5.50	1866 ± 209	4800 ± 172	3.10 ± 0.08	14.23 ± 1.10
D	5.75	1636 ± 63	3266 ± 225	2.24 ± 0.19	8.26 ± 0.62
	One‐way ANOVA	*p* < 0.001	*p* < 0.001	*p* < 0.001	*p* < 0.001
	Tukey–Kramer HSD	AD‐BD‐CD	All except AC	All except AC	All except AC

## 4 DISCUSSION

Pilot hole diameter size plays a significant role in primary screw fixation stability. Many studies have investigated the effect of bone preparation and pilot hole size on the stability screw fixation constructs.^13^, ^17^, ^18^, ^45^ Synthetic bone samples used in experiments have been used to reduce the intervariability among the mechanical properties of samples.^46^, ^47^ The HDPE block can offer a uniform and consistent density that eliminates or lessens the variability and different artifacts during screw insertion, as discussed in ASTM F1717.^48^ The findings of the present study are in agreement with the findings of the existing literature and confirm that pilot hole diameter has a significant impact on the pullout characteristics and IT of the bone screws. We used a novel fixation stability assessment tool, that is, modal analysis, through two different approaches. Both CMA and AMA were used to verify the contradictions between two conventional pullout and IT tests (Figure 3A,B, Figure 5A,B). The acceleration and the sound response were simultaneously recorded after tapping the head of screws by an impact hammer. The recorded data were then used to extract and compare the principal NF of the setup determined from two methods.

In agreement with the study of Turklylmaz et al.,^48^ the increase of the pilot hole diameter from 5 to 5.75 mm (15%), decreased PIT values by 65% due to decreasing resistance induced from material adjacent to the entering threads. Similarly, the S and DE showed a 17% and 72% reduction, respectively, and F_ult_ reduced by 43% as reported by Steeves et al.^16^ Accordingly, IT strength represents the stability of bone screws during screw placement, while pullout characteristics display the stability of bone screw during its removal, however, a few studies have represented contradictory results.^27^, ^49^

Stiffness is the slope of the linear portion of the load–displacement curve and can represent the elastic stability of bone screws in the pullout test. The stiffness “S” decreased by 17% for an increase of 0.25 mm in hole diameter of 5.5 mm. The mean NF determined from both CMA and AMA methods decreased by 3.4% as the pilot hole diameter increased from 5 to 5.75 mm, while that lowered 22% and 17%, for the hole diameter increase from 5.5 to 5.75 mm. Pullout characteristics matched the trend of variations in natural frequencies. The nondestructive modal analysis methods have been proven to quantify the stability of the bone screws,^36^ which present linear stability of the block‐screw structure in its final position that is, final insertion depth. Similarly, S is the linear characteristic of the bone screw stability during screw removal.

All dependent variables that is, the PIT, F_ult_, f_AMA_, and f_CMA_ were significantly different between 5.5 and 5.75 mm pilot hole diameters (Figure 3A,B), which supports the fact that a bigger diameter of the pilot hole was not required. In other words, all measured variables for 5.75 mm pilot hole diameter were significantly lower than those in the other pilot holes. In the pullout test, the maximum DE (or area under the force‐displacement curve) occurred in the 5.5 mm diameter, displaying that the screws in this group size required more work to remove the screw out of the block test. The DE values could be a criterion for primary stability, which is related to both elastic and plastic portions of the force‐displacement curve.^50^

The fixation strength mostly depends on the interface properties for example, prestresses induced from the insertion process, the material properties and contact area between material and bone screw thread.^15^, ^18^, ^20^, ^51^ In every screw insertion, the thread makes the materials deform. The deformation could lead the material to turn into its yield point as a result of prestresses^18^ or even further to shapes a crack at the tip of the screw threads.^15^, ^52^ The length of this crack depends on the ratio of hole diameter per thread diameter, which varied from 77% to 88% in this study. The initial crack length or the number of developed cracks in the 5 mm hole diameter was probably more than that in the other cases. The lowest pilot hole needed the most torque for insertion. As a result, the 5 mm hole diameter has the potential of largest crack length resulting in undesired fracture.

The mechanical screw fixation strength is dependent on the weakest material (the material in which undergoes its yield or postyield behavior) surrounding the screw in the block‐screw interface. The F_ult_, d_ult_, and DE are dependent on the plastic and postyield behavior of materials that were induced next to the screw threads and all were significantly different in this study, except for the 5 and 5.5 groups. Besides, DE is proportional to fracture toughness and the resistance against crack growth. Its results showed that the 5 and 5.5 mm hole diameter acted similarly (Table 2), but it should be noted that more power is needed to insert the screw into the smaller diameter hole. This may locally increase the risk of crack initiation^14^, ^15^ and the probability of the bone fracture.

From a purely geometrical perspective, the allowable load placed on a screw is dependent on the amount of surrounding material that contacts the thread. Therefore, it could be hypothesized that by increasing the contact area between a pedicle screw thread and the surrounding bone, there is a greater distribution of forces and thus larger pullout strength is obtained.^53^ It is obvious that the contact area between polyethylene and bone screw threads decreased by increasing the pilot hole diameter from 5 to 5.75 mm.

Like any other experimental study, the present study suffered from some limitations as follows. First, this study was carried out using HDPE to provide a uniform model and limit the variation encountered in cadaveric bone densities and qualities. The properties of the HDPE may not replicate the exact material characteristics of the anatomical bones. This can affect the accuracy of the measured load and torque data. However, knowing about this limitation, we intended to look at the data by comparing different cases from the qualitative perspective and not quantitative. Second, the optimum parameters related to hole size are limited to the screw designs used in the experiment. The recommended numbers might vary slightly for other screw designs. Third, the ratio of pilot hole diameter over outer diameter (thread diameter) varied only by 11% and thus wider ranges are necessary for more conclusions. Finally, the damage induced in the block‐screw interface has not been quantified directly according to different pilot hole diameters and the damage was speculated based on dependent variables.

## 5 CONCLUSION

First, modal analysis methods that is, classical or acoustic ones can replace the destructive pullout test and have the potential of using in clinics rather than one‐time IT test. Secondly, the size of the pilot hole created prior to screw insertion plays an important role in the stability of bone‐screw fixation. The critical pilot hole size was defined as the size needed to maintain an optimal balance between low IT and high pullout strength. The desired pilot hole diameter has to meet two terms: First, the more contact area between the screw threads and substrate material and second, minimum damage induced in the screw‐block interface. The 5.5 mm pilot hole diameter in this study met these conditions. By performing nondestructive modal analysis and pullout tests on pilot holes with diameters 77%, 81.5%, 85%, and 88% of the bone screw outer diameter being used, the optimal pilot hole diameter was 85% that is, 5.5 mm hole size.

## AUTHOR CONTRIBUTIONS

All authors contributed to the study conception and design. Material preparation, data collection and analysis were performed by Mohammadjavad Einafshar.

Mohammadjavad Einafshar led the study. Ata Hashemi contributed in developing the draft and co‐supervised the study. Ali Kiapour supervised the study, reviewed, and commented on the draft of the manuscript. Ata Hashemi contributed as co‐supervisor and Ali Kiapour contributed in conceptualization of the study. All authors read and approved the final manuscript.

## CONFLICT OF INTEREST

The authors report no conflicts of interest. The authors alone are responsible for the content and writing of this article.
